# Retinal to Retinal Energy Transfer in a Bistable Microbial Rhodopsin Dimer

**DOI:** 10.1021/jacs.5c01276

**Published:** 2025-04-17

**Authors:** Ivo H. M. van Stokkum, Jakub Dostal, Thanh Nhut Do, Lifei Fu, Gregor Madej, Christine Ziegler, Peter Hegemann, Miroslav Kloz, Matthias Broser, John T. M. Kennis

**Affiliations:** † Department of Physics and Astronomy, Faculty of Science, Vrije Universiteit Amsterdam, De Boelelaan 1081, 1081 HV Amsterdam, The Netherlands; ‡ ELI Beamlines Facility, The Extreme Light Infrastructure ERIC, Za Radnicí 835, 25241 Dolní Břežany, Czech Republic; § Department of Structural Biology/Biophysics II, University of Regensburg, DE-93053 Regensburg, Germany; ∥ Institut für Biologie, Experimentelle Biophysik, Humboldt-Universität zu Berlin, Invalidenstr. 42, D-10115 Berlin, Germany

## Abstract

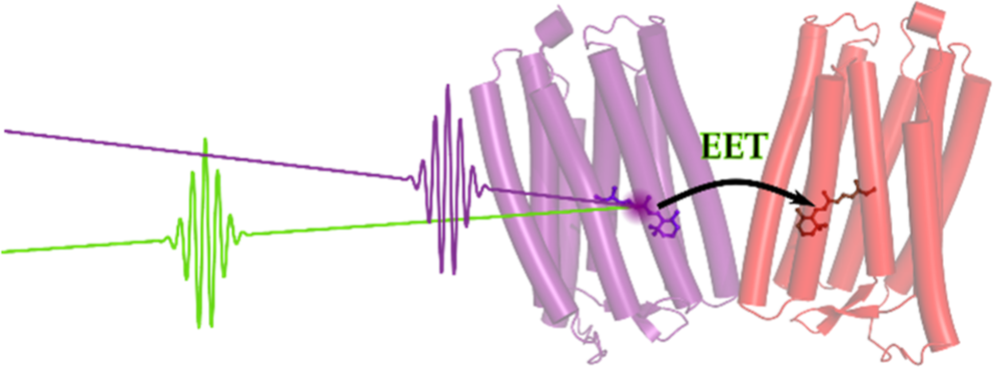

Neorhodopsin (NeoR) is a newly discovered fungal bistable rhodopsin that reversibly photoswitches between UV- and near-IR absorbing states denoted NeoR_367_ and NeoR_690_, respectively. NeoR_367_ represents a deprotonated retinal Schiff base (RSB), while NeoR_690_ represents a protonated RSB. Cryo-EM studies indicate that NeoR forms homodimers with 29 Å center-to-center distance between the retinal chromophores. UV excitation of NeoR_367_ takes place to an optically allowed S3 state of 1B_u_^+^ symmetry, which rapidly converts to a low-lying optically forbidden S1 state of 2A_g_^–^ symmetry in 39 fs, followed by a multiexponential decay to the ground state on the 1–100 ps time scale. A theoretically predicted nπ* (S2) state does not get populated in any appreciable transient concentration during the excited-state relaxation cascade. We observe an intradimer retinal to retinal excitation energy transfer (EET) process from the NeoR_367_ S1 state to NeoR_690_, in competition with photoproduct formation. To quantitatively assess the EET mechanism and rate, we experimentally addressed and modeled the EET process under varying NeoR_367_-NeoR_690_ photoequilibrium conditions and determined the EET rate at (200 ps)^−1^. The NeoR_367_ S1 state shows a weak stimulated emission band in the near-IR around 700 nm, which may result from mixing with an intramolecular charge-transfer (ICT) state, enhancing the transition dipole moment of the S1–S0 transition and possibly facilitating the EET process. We suggest that EET may bear general relevance to the function of bistable multiwavelength rhodopsin oligomers.

## Introduction

Microbial rhodopsins are photoreceptor proteins that sense light via a protonated retinal Schiff base (RSB) chromophore.^1^ They form important tools for optogenetics, a research field that aims to control brain and cellular function noninvasively by means of light.^2^ In addition, microbial rhodopsins have been engineered to serve as optical voltage sensors, enabling all-optical visualization and quantification of neuronal action potentials.^3^ Recently, a great interest in far-red-shifted microbial rhodopsins has arisen, in particular with the discoveries of NeoRhodopsin (NeoR)^4^ and Bestrhodopsins.^5,6^ NeoR is a newly discovered fungal rhodopsin-cyclase with unusually red-shifted narrowband absorption peaking at 690 nm.^4,7−10^ Its strongly fluorescent rhodopsin-module has great promise for application in optogenetics and neuroimaging.

**Figure 1:**
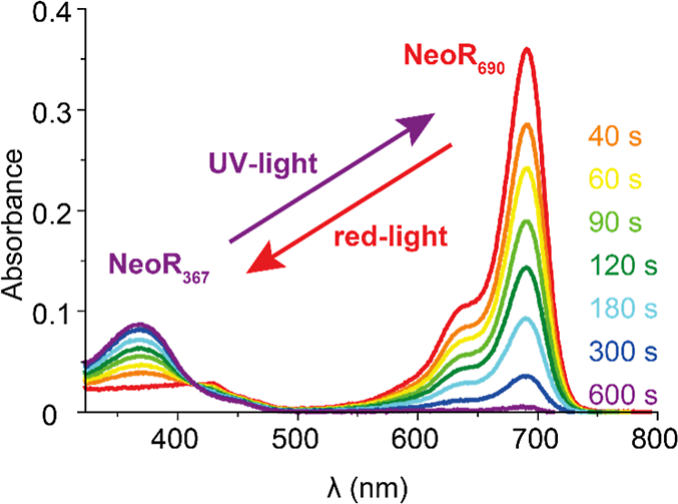
Absorption spectra showing the progressive photoconversion of the red-absorbing state (NeoR_690_) into the near UV-absorbing state at 367 nm (NeoR_367_) by red illumination. Total illumination time for each spectrum as indicated, illumination was at 690 nm at 13 mW/cm^2^. (Figure adapted from,^4^ available under a CC-BY-4.0 license. Copyright 2020 M. Broser et al.).

**Scheme 1:**
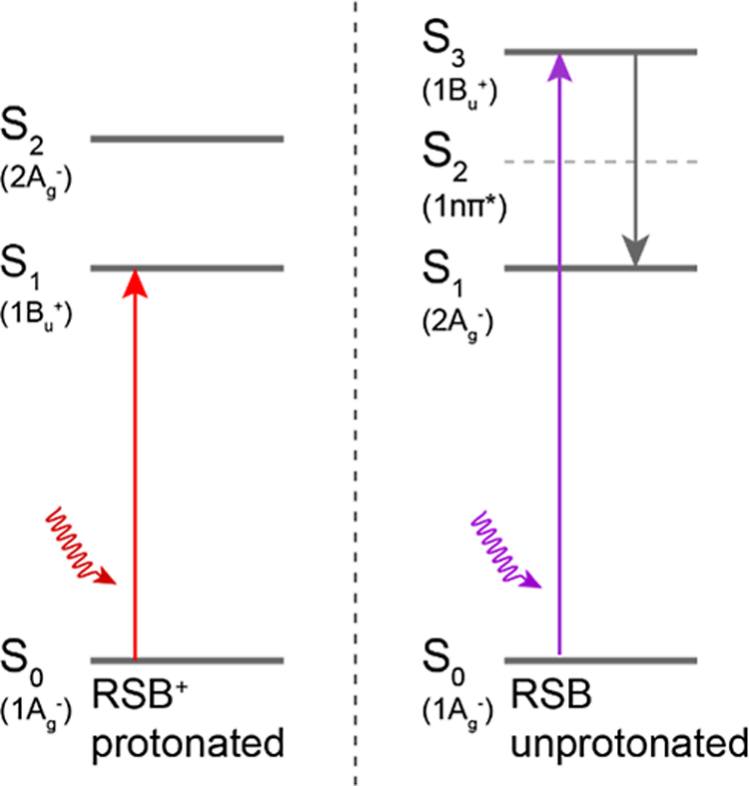
Energy Level Ordering in Protonated RSB (Left Panel) and Unprotonated RSB (Right Panel)

Upon application of red light, NeoR converts from a red-absorbing state (NeoR_690_) with an all-trans RSB^4^ to a highly unusual *7-cis* UV state absorbing maximally at 367 nm^10^ (NeoR_367_) (Figure 1). Subsequent UV-A light illumination converts NeoR_367_ back to NeoR_690_. Stable UV-absorbing states rarely appear in microbial rhodopsins and have only scarcely been investigated.^7,9,11−16^ Moreover, many UV-absorbing animal rhodopsins exist that have not been accessible for spectroscopy due to poor recombinant expression.^17^ In visible-light absorbing rhodopsins including NeoR_690_, the RSB is protonated and light absorption promotes the chromophore to the lowest excited state, S1, with B_u_^+^ symmetry designated as 1B_u_^+^, with an excited state of A_g_^–^ symmetry (2A_g_^–^, S2) energetically above it (Scheme 1).^18^ In contrast, in UV-absorbing rhodopsins the RSB is unprotonated and as a consequence the excited-state manifold is energetically rearranged: the 1B_u_^+^ state is the optically allowed light absorbing state, designated here as S3, while a lower-lying excited state of A_g_^–^ symmetry is optically forbidden, designated 2A_g_^–^ or S1.^12,18,19^ Because of the lone electron pair at the nitrogen heteroatom, an nπ* state is likely to exist in the excited-state manifold, tentatively designated here as S2 (Scheme 1) and predicted to be almost degenerate with the optically bright state^20^ or above it.^21^

Upon light absorption, the 1B_u_^+^ state rapidly relaxes to lower-lying excited states in unprotonated RSB.^12,22,23^ In Histidine-Kinase-Rhodopsin-1 (HKR1) from *Chlamydomonas reinhardtii*, such unusual photophysics was experimentally observed for the UV-absorbing HKR1 involving the optically forbidden 2A_g_^–^ excited state below a bright optically allowed 1B_u_^+^ excited state.^12^ Here we present the ultrafast photoreactions of the UV-sensitive chromophore in NeoR_367_ by means of ultrafast transient absorption (TA) and femtosecond stimulated Raman spectroscopy (FSRS). We find that as in HKR1, the lowest excited state of NeoR_367_ corresponds to the 2A_g_^–^ state and describe an excitation energy transfer (EET) process between adjacent NeoR_367_ and NeoR_690_ retinal molecules where the 2A_g_^–^ state acts as an energy donor.

## Results and Discussion

### Observation of Ultrafast 1B_u_^+^–2A_g_^–^ Conversion and Intradimer Excitation Energy Transfer

**Figure 2:**
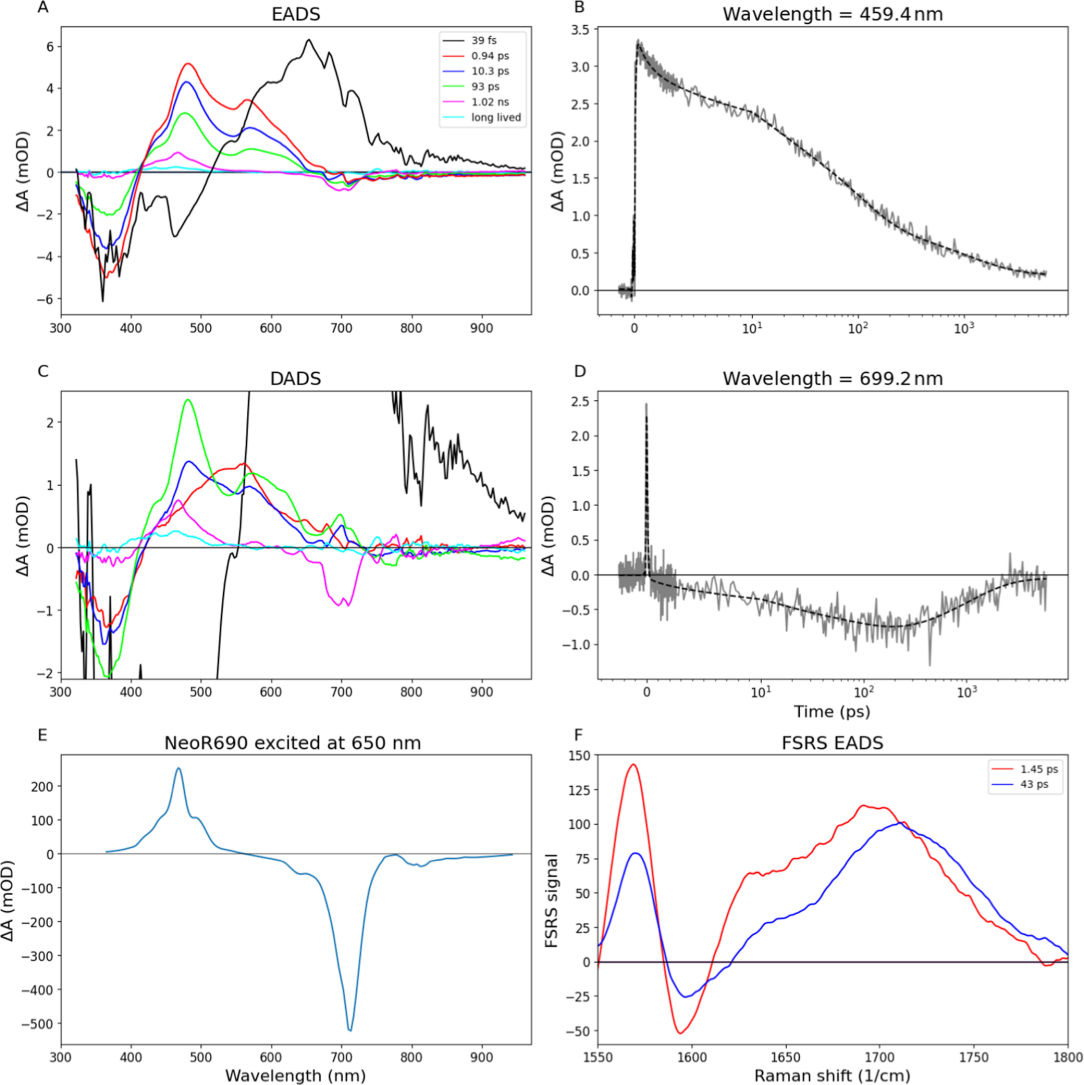
Evolution-associated difference spectra (EADS) (A) and Decay-associated difference spectra (DADS) (C) estimated with a sequential or parallel analysis of NeoR_367_ upon excitation at 360 nm. (B,D) Representative traces of data (gray) and fit (black dashed). Note that the time axis is linear until 10 ps, and logarithmic afterward. The estimated lifetimes are 39 fs (black), 0.94 (red), 10 (blue), 93 ps (green), 1.0 ns (magenta) and long-lived (cyan). For presentation purposes the black EADS has been divided by 2. (E) Time-gated spectrum at 100 ps of NeoR_690_* with direct excitation at 650 nm; (F) FSRS EADS of the NeoR_367_ S1 state.

We performed an ultrafast TA experiment^24^ on NeoR_367_, which was accumulated through red background illumination. Given the very low quantum yield of ≈0.001 for NeoR_690_ to NeoR_367_ photoconversion,^9^ moderately strong background illumination of 9 mW/cm^–1^ was applied to convert the sample to NeoR_367_ (see Methods). Because of the absence of thermal reversion of NeoR_367_,^4^ only the actinic laser (20 μW) converts the sample back to NeoR_690_ and hence the moderate illumination conditions could be maintained during the experiments. The fs-laser excitation wavelength was 360 nm and the absorbance changes were recorded between 320 and 960 nm. 1000 time points were taken, thereby finely sampling the signals around time zero. In addition, the coherent/cross phase modulation artifact was recorded separately in a buffer sample and subtracted from the data. As a result, very short-lived components could be extracted from the data with high confidence. The transient data were globally analyzed with a kinetic scheme with sequentially interconverting intermediates (Figures 2B,D and S1). Note that the spectral evolution of the molecular species does not necessarily occur sequentially, and that the sequential analysis provides a first qualitative assessment of the data. A target analysis involving particular kinetic schemes with specific connectivities is presented in a later section of the manuscript. Five time-constants of 39 fs, 0.94 ps, 10 ps, 93 ps, 1.0 ns and a long-lived component were required for a satisfactory fit. Figure 2A shows the evolution-associated difference spectra (EADS). The first EADS (black line) shows a ground-state bleach (GSB) near 360 nm, a stimulated emission (SE) band at 470 nm and a broad excited-state absorption (ESA) band around 650 nm, indicating population of the 1B_u_^+^ state (S3). The observation of SE at 470 nm provides definite proof that it is optically allowed, whereas its rapid relaxation to the lower-lying S1 state proves that it is not the lowest excited state. Its lifetime of 39 fs and spectral shape are very similar to that observed previously for HKR1 UV rhodopsin.^12^ Here, the NeoR_367_ S3 lifetime (39 fs) is assigned with significantly higher confidence than for HKR1 (where it was assigned as <100 fs)^12^ given the finer sampling around time zero and the separate recording and subtraction of the coherent artifact.

The S3 state evolves in 39 fs to the next EADS (red line), which shows a GSB around 360 nm and ESA bands at 480 and 580 nm. Given its spectroscopic signature it may be assigned to population of the lower-lying 2A_g_^–^ (S1) excited state, as observed in HKR1 UV rhodopsin.^12^ Depopulation of the S1 state is observed with lifetimes of 0.94 ps, 10 and 93 ps given that the corresponding EADS have similar spectral shape at decreasing amplitude. The 0.94 ps component may also include a vibrational cooling component considering that the red wing at 600 nm loses relatively more amplitude than the GSB. In HKR1, the multiexponential decay of the S1 state was assigned to distinct populations with different reactivity.^12^ The NeoR S1 dynamics likely represents complex motion of several population fractions on a multidimensional potential energy surface that mostly (≈90%) recombine to the original NeoR_367_ ground state. The nondecaying EADS (cyan) corresponds to a long-lived photoproduct that absorbs at 460 nm: its nature will not be discussed here but will be the topic of a forthcoming paper.

Prior to the appearance of the long-lived photoproduct, the fourth EADS evolves to the fifth EADS (magenta line) in 93 ps. Strikingly, this EADS shows a negative signal around 700 nm, a positive signal at 460 nm and has a lifetime of 1.0 ns, indicating that it does not correspond to the S1 state of NeoR_367_. Rather, its spectrum and lifetime closely resemble the NeoR_690_ excited state (NeoR_690_*) which has its GSB/SE and ESA at these wavelengths.^4^Figure 2E shows a time-gated spectrum of NeoR_690_* with UV background illumination and excitation at 650 nm recorded on the same setup, demonstrating beyond doubt that the species represented by the fifth EADS corresponds to NeoR_690_*. Figure 2D shows the kinetics at 700 nm. The signal starts at a negative amplitude after zero delay and increases in amplitude on the 93 ps time scale, after which it decays again in 1 ns. The positive amplitudes of the blue and green decay-associated difference spectra (DADS) around 700 nm indicate an increase of the negative amplitude which is clearly visible in Figure 2C. Hence, the NeoR_690_* excited state is formed dominantly in 93 ps upon initial population of the NeoR_367_ excited states. This delayed time evolution excludes the possibility of a direct population of the NeoR_690_* excited state by the exciting UV laser and strongly suggests that excitation energy transfer (EET) occurs from the NeoR_367_ S1 state to the NeoR_690_ state. This can only be understood if one assumes that a small fraction of NeoR_690_ remains in the sample due to the incomplete conversion to NeoR_367_, and that the donor and acceptor chromophore are sufficiently close.

FSRS spectroscopy^25−27^ was applied to further characterize the nature of the NeoR_367_ excited states. Figure 2F shows the FSRS EADS of the S1 state that follow from a sequential analysis, with lifetimes of 1.45 and 47 ps. They exhibit an upshift of the C=C ethylenic stretch to ∼1710 cm^–1^, which is a highly specific marker band for the 2A_g_^–^ state,^12^ providing further evidence that the NeoR_367_ S1 state has 2A_g_^–^ character. Here, we resolved two time constants (1.45 and 47 ps) rather than the three in the TA data (0.94, 10, and 90 ps) which is due to sparser temporal sampling and a lower signal-to-noise ratio. The TA and FSRS data provide no evidence for transient population of the 1nπ* (S2) state: an in-depth discussion of the ordering of the 1B_u_^+^_,_ 2A_g_^–^ and 1nπ* states and their interrelation will be given in a separate section of the manuscript. Figure S2 shows kinetic traces at selected wavenumbers along with the fit result. The data could be well described with three lifetimes of 100 fs, 1.45 and 47 ps and a nondecaying component, but a strong FSRS coherent artifact around time zero did not allow to properly resolve the 39 fs S3–S1 internal conversion process. The long-lived component is not shown.

### Cryo-EM Microscopy Reveals an Unusual NeoR Dimeric Arrangement

**Figure 3:**
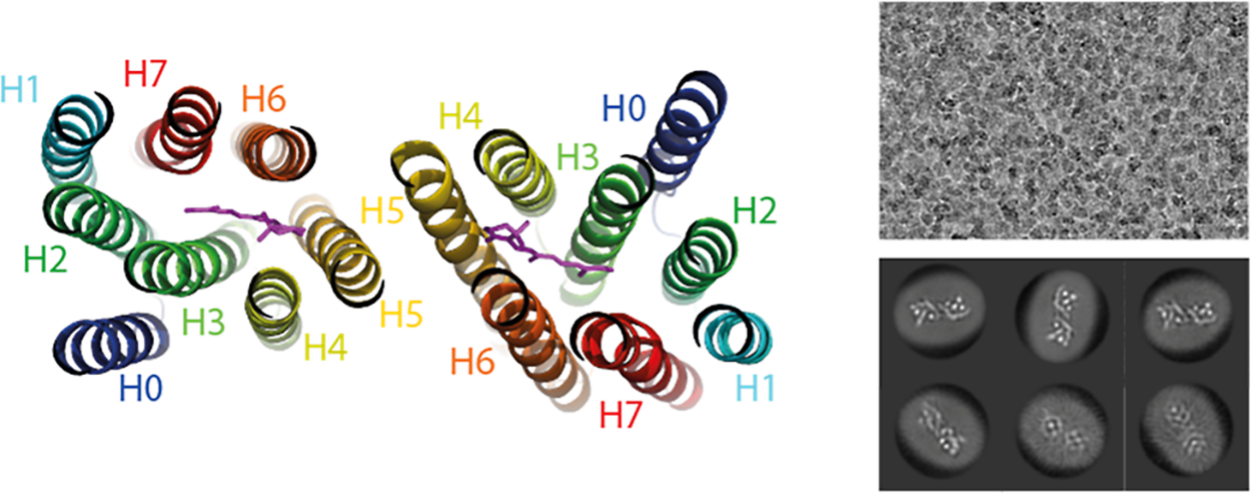
Homodimeric arrangement of the NeoR sample as derived from CryoEM. Helices are numbered according to canonical rhodopsins (H1–H7), with the preceding extra helix (H0) found for enzymerhodopsins. Right: representative CryoEM micrograph and selected 2D-class averages.

To establish a more solid foundation for interpreting the observed EET process, we conducted a detailed characterization of the NeoR rhodopsin sample used in spectroscopy, including single-particle cryo-electron microscopy (CryoEM). Size-exclusion chromatography revealed a monodisperse sample in a single oligomeric state, confirmed as a homodimer through motion-corrected micrographs obtained by CryoEM (Figures 3 and S3). The final map at 4.8 Å resolution shows the locations of the transmembrane α-helices and allows for fitting the overall dimeric arrangement of the two NeoR rhodopsins using a structural model derived from Alphafold2 (AF2).^28^ When modeling the protein by AF2, the retinal chromophore was not considered. However, the chromophore binding pocket microbial rhodopsins including NeoR are highly conserved, despite the different spectral properties. The retinal positions were taken from structural superposition with the crystal structure of Rhodopsin-Phosphodiesterase.^29^ Hence, this approach provides a reliable assignment of the general localization of the retinals.

The resulting homodimeric structure is shown in Figure 3. In this arrangement, the NeoR monomers are oriented such that Helix 5 is positioned closest to the C2-axis of the dimer, with both β-ionone rings of the retinal chromophores facing inward toward each other. The retinal center-to-center distance is approximately 29 Å, while the edge-to-edge distance of the β-ionone rings is around 19 Å. This homodimeric organization contrasts with that observed in Rhodopsin-Phosphodiesterase (RhPDE), a related enzymerhodopsin with known crystal structure (PDB: 7CJ3).^29^ In RhPDE, homodimerization occurs via interactions between Helix 1 and Helix 7, with the RSB facing the dimeric center. A similar interface is predicted for functional rhodopsin-guanylyl cyclases [RGCs (Figure S3E,F)].

### Demonstration of Intradimer EET in Dimers of Variable UV-Red Composition

**Figure 4:**
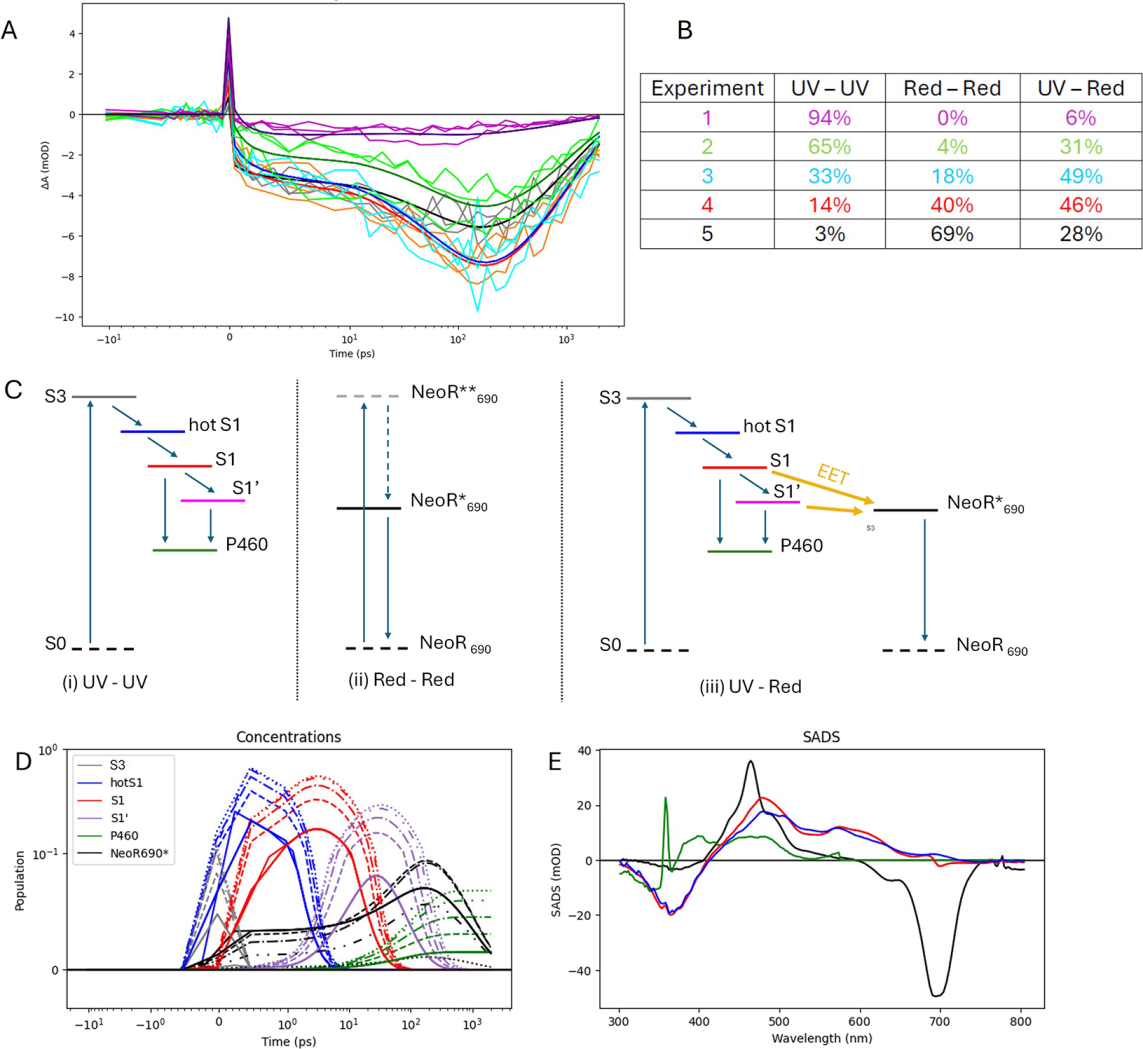
(A) Transient absorption of NeoR in five conditions monitored at 711 nm upon excitation at 360 nm. Key: experiment 1 (magenta), 2 (green), 3 (cyan), 4 (orange), 5 (gray). Black, red, blue, dark green and purple lines indicate the target analysis fit. (B,C) Dimer fractions (with color key from A) and schematic depiction of the target analysis kinetic schemes for (i) UV–UV dimers; (ii) Red–Red dimers; (iii) UV-Red dimers. Color key: gray S3, blue hot S1, red S1, purple S1′, green P460 photoproduct; black NeoR_690_* excited state. For case (i), transitions from hot S1, S1 and S1′ to S0 have not been depicted. In case (ii), excitation occurs to a higher excited state NeoR**, which rapidly relaxes to the lowest excited state NeoR*. NeoR** is not further considered in the target analysis. (D) Populations of the states with the color code indicated in (C) that follow from the target analysis under the 5 excitation conditions. Line type key: 1 (dotted), 2 (dot dot dashed), 3 (dot dashed), 4 (dashed), experiment 5 (solid). (E) SADS estimated from the target analysis, with color code identical to panel (D). Note that the SADS of S1 and S1′ are assumed identical and are indicated in red. For presentation purposes the black NeoR_690_* SADS has been divided by 2, and the S3 SADS has been omitted in panel (E). Both axes in panel (D) are partially linear, and partially logarithmic.

For the EET process to occur, individual NeoR homodimers need to exist in a mixed NeoR_367_–NeoR_690_ state, hereafter referred to as UV-Red. This implies that the yield of the process should depend on the intensity of the background illumination, which determines the ratio of NeoR_367_ and NeoR_690_ in the sample. The concentration of UV-Red homodimers then follows from a binomial distribution that defines the ratio of NeoR_367_–NeoR_367_, hereafter referred to as UV–UV, NeoR_690_–NeoR_690_, hereafter referred to as Red–Red, and UV-Red. To further validate the EET hypothesis, we performed a series of 360 nm excitation TA experiments with varying red background illumination, resulting in distinct concentration ratios of NeoR_367_ and NeoR_690_. The sample absorption was monitored during the TA experiments in situ using the detector array of the TA setup, by recording probe-only absorption measurements in between TA acquisitions (Figure S4A). The resulting absorption spectra were decomposed into a NeoR367 and NeoR690 component, enabling quantitative determination of the relative amounts of NeoR_367_ and NeoR_690_ and hence the relative concentrations of UV–UV, Red–Red and UV-Red dimers in an empirical fashion (Figures 4B and S4E,F).

Figure 4A shows the kinetics at 711 nm monitoring the rise of NeoR_690_* under five red background illumination intensity conditions, referred to as experiments 1–5. In the case of the high background illumination (experiment 1, magenta curve) nearly all dimers (94%) are in the UV–UV state and the EET process barely occurs, consistent with the overall low signal amplitude. Upon decreasing the background illumination, the relative concentration of UV-Red dimers increases to 31% and accordingly the signal amplitude becomes larger (experiment 2, green curve), showing the rising NeoR_690_* signature on the 100 ps time scale. An additional decrease of background illumination lifts the UV-Red dimer concentration to its maximal value (49% and 46% respectively), and indeed the signal amplitude is maximized at these conditions (experiment 3, cyan and experiment 4, orange curves). Note that under these conditions, a significant fraction of directly excited NeoR_690_* is observed as well, as indicated by the prompt rise of the signal at time zero and follows from the small absorption of NeoR_690_ at 360 nm (Figure 1). At the lowest background illumination intensity, the Red–Red dimers become dominant, and the signal amplitude consequently decreases (experiment 5, black curve). Additional kinetic traces are shown in Figure S5.

In a simultaneous target analysis^30^ of these five data sets, we described the data at each illumination condition as a superposition of (i) the pure NeoR_367_ state (UV–UV), described by a sequential scheme with 5 states that includes the S3 state, a hot S1 state, the relaxed S1 and S1′ states and the product P460. Note that S1 and S1′ were assumed to have identical spectral shapes and the relaxation of S1 to S1′ denotes partial population decay to the ground state on the 10 ps time scale. (ii) The pure NeoR_690_ state (Red–Red), approximated by a single exponential decay and taken into account as a directly excited fraction of NeoR_690_* in the data, and (iii) the Dimer state UV-Red, i.e. NeoR_367_–NeoR_690_, in which EET channels have been added from S1 to NeoR_690_, as schematically depicted in Figure 4C. The full kinetic model is given in Figure S6, while the fractions of (i), (ii) and (iii) in the five data sets are indicated in Figure 4B. The darker colored lines in Figures 4A and S5 denote the fit result of the target analysis. The target analysis yields species-associated difference spectra (SADS), which represent the spectra of the “pure” molecular states, while EADS may consist of mixtures of molecular states.^31^

The populations and SADS estimated from the target analysis are depicted in Figure 4 D,E. For case (i) UV–UV, S3 (gray) decays with a lifetime of 39 fs to hot S1 (blue). With a lifetime of ≈1 ps hot S1 partially decays to GS and relaxes to S1 (red). Subsequently, with a lifetime of ≈12 ps most of S1 decays to GS and to S1′ (purple) which decays with a lifetime of ≈113 ps, partially forming a long-lived product P460 (dark green). Note that S1 and S1′ are assumed to have the same SADS, and hence only a red curve is shown. For case (ii) Red–Red, NeoR_690_ is directly excited to state NeoR_690_* (black) with 360 nm, its absorption relative to that of NeoR_367_ (UV) is ≈4%. Accordingly, the NeoR_690_* state shows a GSB around 360 nm, confirming the possibility of direct absorption. For case (iii) UV-Red, we estimated an EET rate from S1 to NeoR_690_ of (200 ps)^−1^.

Hence, we have quantitatively described the series of five power excitations with a single set of SADS and a common kinetic model, thereby only varying the relative initial populations of UV–UV, UV-Red and Red–Red dimers, to finally estimate an EET rate from S1 to NeoR_690_ of (200 ps)^−1^. This result also indicates that in UV-Red dimers, EET competes with P460 product formation, as can be seen in Figure 4D: whereas for low background illumination the relative population of NeoR_690_* is high (solid black line) and that of P460 is low (solid green line), for high background illumination the relative population of NeoR_690_* is low (dotted black line) and that of P460 is high (dotted green line).

We applied the same kinetic scheme to the TA data of Figure 2, the results are shown Figures S7 and S8. The estimated fraction NeoR_690_ was 10%, resulting in SADS that were consistent with the results of Figure 4, cf. Figures 4E and S7, and explaining the origin of the instantaneous negative signal at 700 nm in Figure 2D.

### Nature of the NeoR_367_ S1 Excited State

We have assigned the NeoR_367_ S1 state to an optically forbidden excited state below the optically allowed S3 state. Because the S1 state acts as the energy donor to NeoR_690_ we discuss its nature in more detail. First and foremost, we note that the precise molecular identity of the unprotonated RSB S1 state so far has only been sparsely studied experimentally^12,22,23,32^ and even less so theoretically and computationally.^18,20,21^ Contrary to our experimental observations, the 2A_g_^–^ state was predicted to lie slightly above the 1B_u_^+^ state in ref (21) and was not predicted in ref (20).

Most microbial rhodopsins bind a protonated RSB, for which it is known that the S0 ground state has A_g_^–^ symmetry and is hence labeled as 1A_g_^–^, also known as a covalent state so as to indicate that the charges are distributed evenly over the conjugated π-electron system.^33^ This is true for the dark-adapted states as well as for all photocycle intermediates. The S1 state of protonated RSB has B_u_^+^ symmetry, also known as an ionic state to indicate that charges accumulate at specific sites of the π-electron system. Because of the different symmetries of the involved state, the S0–S1 optical transition is strongly allowed (Scheme 1). The S2 state, labeled 2A_g_^–^, has A_g_^–^ symmetry just as the S0 state and consequently is optically dark. In some microbial rhodopsins mixing of the S1 and S2 states occurs, which is important for RSB reactivity and fluorescence.^34−37^

The situation in microbial rhodopsins with an unprotonated RSB is significantly different. Experiments on unprotonated retinal Schiff base in solution, which absorbs near 360 nm show that weak emission occurs at 520–600 nm, which implies that the Stokes shift amounts to 8000–11,000 cm^–1^.^22,23^ This observation demonstrates beyond doubt that the S1 state is not the absorbing state because such a large Stokes shift from the same absorbing and emitting excited state is unphysical. An early theoretical treatise proposed that as compared to the protonated RSB, the energy ordering in the unprotonated RSB of 1B_u_^+^ and 2A_g_^–^ states was reversed, and hence the 2A_g_^–^ state could be identified as the S1 state (Scheme 1).^18^ It is well established in linear polyenes that the 1B_u_^+^–2A_g_^–^ energy gap is sizable and assumes values to thousands of cm^–1^^38^ and may lead to such large Stokes shifts. The radiative lifetime, which is a measure for the strength of an optical transition,^39^ of the unprotonated RSB in solution amounts to 250–350 ns, which indicates an optically forbidden nature expected for S0 and S1 states of the same symmetry (i.e, A_g_^–^).^22,23^ For comparison, the S1 state in canonical microbial rhodopsins with protonated RSB has a radiative lifetime of 6 ns,^40^ which implies that its S1–S0 transition is ≈50 times stronger. The UV–visible spectral signature of the S1 state in NeoR_367_ (Figures 2A and 4E) and HKR1^12^ is similar to that of the unprotonated RSB in solution,^32^ indicating similar molecular natures.

Importantly, because the unprotonated RSB has a lone electron pair on its nitrogen heteroatom, a nπ* state is expected in the excited-state manifold,^20,21^ similar to retinal which has two lone electron pairs on its oxygen heteroatom.^41^ It is instructive to compare the excited-state relaxation dynamics of unprotonated RSB in NeoR and HKR1 with those of retinal in solution. The excited-state dynamics of retinal in apolar organic solvent has been well characterized: upon UVA light absorption by the 1B_u_^+^ state (S3), rapid internal conversion takes place in 40 fs to the 2A_g_^–^ state (S2), which converts to the 1nπ* state (S1) in 400 fs, which finally populates the triplet state at high yield in 30 ps through intersystem crossing.^42−45^ Hence, in retinal in apolar solvent the 1nπ* forms the lowest excited state and is designated the S1 state. Notably, the UV–vis spectroscopic signatures of the retinal 1B_u_^+^ and 2A_g_^–^ states are very similar to those of unprotonated RSB in NeoR and HKR1:^12^ the 39 fs EADS (Figure 2A) matches that of retinal 1B_u_^+^, while the 0.94, 10, and 93 ps EADS (Figure 2A) match that of retinal 2A_g_^–^.^42,46^ In addition, the triplet signatures of retinal and the unprotonated RSB are similar.^32,42,46^ However, no matching signature of the retinal 1nπ* state, which has a weak absorption around 450 nm,^42,46^ is discernible in the EADS of NeoR or HKR1. Instead, FSRS spectroscopy indicates an unusually upshifted RSB C=C ethylenic stretch mode at ≈1710 cm^–1^ in the S1 state of both NeoR (Figure 2F) as in HKR1,^12^ which provides strong evidence for population of the 2A_g_^–^ state. Such upshifts are a well-established phenomenon in linear polyenes and carotenoids^26,47−53^ and arises from a strong vibronic coupling between the 1A_g_^–^ and 2A_g_^–^ states.^54^ Similar upshifts were observed for an unprotonated RSB analogue in solution.^55^ We can exclude that the 1nπ* state would have an upshifted C=C stretch band similar to the 2A_g_^–^ state, because retinal in apolar solvent does not show such an upshifted C=C band while it resides in the 1nπ* state on the picosecond time scale.^45^ We conclude that the 1nπ* state does not get transiently populated in NeoR and HKR1 and that it most likely is energetically higher than the 2A_g_^–^ state. Hence, we can assign the 2A_g_^–^ state as the lowest excited state, S1. Nevertheless, the 1nπ* state is likely present in the unprotonated RSB excited-state manifold given that in organic solvent the S1 state evolves to the triplet state through intersystem crossing,^32^ which is only possible from a state involving lone electron pairs.^20^ The triplet yield in unprotonated RSB in solution is very low (<5%)^32^ as compared to retinal in apolar solvent (74%),^42^ which suggests a thermally activated 2A_g_^–^ → 1nπ* → triplet formation, which is consistent with our conclusion that the 2A_g_^–^ state lies energetically lower than the 1nπ* state. It also suggests that the 1nπ* state is significantly lower than the 1B_u_^+^ (S3) state, otherwise no thermal activation of the triplet state would be possible (Scheme 1). In ref (21) the 1nπ* state was estimated above 2A_g_^–^, as well as above 1B_u_^+^.

Notably, the NeoR_367_ S1 SADS (Figure 4E and Figure S7C, blue and red lines) show evidence of weak stimulated emission above ≈700 nm, which is absent in the S1 TA signals of unprotonated RSB in apolar organic solvent.^32^ This observation implies that the NeoR_367_ S1 state in fact has a detectable transition dipole moment with the S0 ground state and hence is not entirely optically forbidden. Similar results were observed for HKR1.^12^ This phenomenon is strikingly similar to that observed for retinal in polar solvent,^46^ where it was proposed that an intramolecular charge-transfer (ICT) state was sufficiently stabilized by the polar environment to mix with the 2A_g_^–^ state to enhance its transition dipole moment and result in stimulated emission. For carbonyl substituted carotenoids such as peridinin, fucoxanthin, spheroidenone and echinenone^56−58^ it is well established that as a result of the electronegativity of the substituted oxygen, such state mixing occurs in polar solvents,^59−61^ photosynthetic light harvesting complexes^62−64^ and the orange carotenoid protein (OCP).^65,66^ This 2A_g_^–^/ICT mixing partially lifts the optical forbidden nature of the S1–S0 transition and results in (weak) stimulated emission in the red or near-infrared regions. Given its electronegativity, the nitrogen heteroatom of the unprotonated RSB in NeoR possibly plays a similar role in stabilizing an ICT state under the influence of a polar environment around the Schiff base.

### Nature of the EET Process

Our CryoEM data show that the dimerization interface in the NeoR sample is arranged in such a way that a 29 Å center-to-center distance results between adjacent chromophores. Although the 4.8 Å map is insufficient to directly detect the retinal chromophore, the highly conserved architecture of the retinal binding pocket in microbial rhodopsins allows for a reliable estimation of chromophore localization within the homodimeric complex. Even if in the AlphaFold2 model some slight deviations in the mutual retinal positioning might occur, these are not expected to have an appreciable effect on the EET rate. With a 19 Å edge-to-edge distance between the RSBs, the point-dipole approximation which defines simple Förster theory and requires that the distance between chromophores must significantly exceed their size is not strictly valid. Instead, more advanced methods that take into account the charge distributions on the donor and acceptor molecules in ground and excited states need to be applied.^67−70^

EET phenomena involving optically forbidden states are well-known from natural and artificial photosynthetic light harvesting antennae, in particular in the light harvesting and photoprotective functions of carotenoids. Carotenoids have an electronic structure similar to that of the unprotonated RSB except for the absence of a low-lying nπ* state, with an optically allowed S2 state (1B_u_^+^) and an optically forbidden S1 state (2A_g_^–^) that generally lie lower in energy as compared to the unprotonated RSB due to their longer π-electronic conjugation length.^56^ Carotenoids are usually bound to light harvesting complexes in close proximity to (bacterio)chlorophylls, and in some cases transfer their energy to chlorophyll via the S1 state^51,56,71−74^ or accept energy from excited-state chlorophyll to the S1 state.^52,75−77^ The exact mechanisms by which these processes occur are still under discussion, but there is general agreement that in unsubstituted carotenoids, symmetry breaking due to backbone distortions that result from protein binding lead to a mixing of the S2 and S1 states, conferring some transition dipole moment to the S1–S0 transition,^69,70,78−80^ even if quantitative agreement with experiments is controversially discussed.^69^ In addition, in carbonyl-substituted carotenoids mixing of the 2A_g_^–^ state with ICT states significantly contributes to EET efficiency in photosynthetic light harvesting through enhancement of the transition dipole moment.^62,81^ In NeoR_367_, the low-amplitude S1 stimulated emission band at 700 nm (Figure S6C) may follow from either of the mechanisms described above or a combination thereof. With the gained S1–S0 transition dipole moment and significant spectral overlap with the NeoR_690_ ground state absorption, the EET process is likely enhanced. Even if Förster theory is not strictly applicable, we conducted a structure-based Förster EET calculation as outlined in the Supporting Information. In the absence of emission data from NeoR_367_, we took the fluorescence spectrum and the radiative lifetime (250 ns) of the unprotonated RSB in organic solvent as an approximation. We arrived at a EET rate constant of (1 ns)^−1^, which is a factor of 5 slower than the experimentally observed EET rate constant of (200 ps)^−1^. This result suggests that within the accuracy of the FRET calculation, the radiative lifetime of the S1 donor state needs to be shortened by a factor of 5, and hence the transition dipole moment needs to be enhanced, with respect to that of unprotonated RSB in organic solvent to enable the EET process.

In most photosynthetic light harvesting antennae, EET processes occur at close carotenoid-chlorophyll proximity of ≈10 Å. A striking recent example of EET involving optically forbidden states at center-to-center distances comparable to those reported here (29 Å) was recently provided by the quenched phycobilisome-OCP complex of cyanobacteria,^67,82^ where advanced time-resolved spectroscopic experiments combined with extensive target modeling revealed that bilin chromophores in the phycobilisome transfer their excited-state energy to the optically forbidden S1/ICT state of an OCP fragment-bound canthaxanthin on a 100 ps time scale.^83^ This newly characterized process was rationalized through structure-based quantum-chemical computational methods, reproducing the experimental results.^67^ Similar advanced structure-based computational methods including a rigorous account of the unprotonated RSB excited-state electronic structure bound to NeoR in a 7-*cis* configuration will be required to quantitatively describe the EET processes reported here.

The observation of a retinal–retinal EET process in rhodopsins is reminiscent of the carotenoid-retinal EET process in Xanthorhodopsins (XRs)^84^ and recently discovered similar carotenoid-binding retinal proton pumps.^85^ XRs bind a carotenoid to enhance the absorption cross section by harvesting blue photons, and transferring the excited-state energy to the RSB through EET,^86−89^ followed by RSB isomerization. The carotenoid is bound to the rhodopsin surface in a so-called fenestration cleft which brings it in close contact with the RSB, allowing reasonably efficient EET.^90^ We note that the conditions for EET are vastly different for XRs and NeoR: in the former case, the carotenoid’s conjugated π-electron system (its polyene backbone) must be in very close contact with that of the RSB because EET occurs exclusively from the carotenoid’s optically allowed S2 (B_u_^+^) state,^86,89^ which has an intrinsic lifetime of only ≈150 fs, after which it internally converts to the optically dark S1 state (2 A_g_^–^)^56^ which itself is too low in energy to participate in the EET process.^86,89^ Hence, the EET process must occur at a rate in the same order of ≈150 fs in order to compete with S2–S1 internal conversion, and EET hence proceeds at rates that are ≈1000 fold faster than that observed here for NeoR. This kinetic limitation is the reason for the existence of the fenestration cleft in the XR structures: it allows carotenoid binding at a short distance of 11 Å to the retinal in an orientation that is conducive for extremely fast EET.

### Significance of EET in Oligomeric Rhodopsin Arrangements

The observed EET process occurs within a homodimeric arrangement which appears unphysiological, as NeoR functions exclusively in vivo as part of a heterodimeric complex.^4^ Thus, our characterization presented here should be regarded as a proof of principle, demonstrating the fundamental biophysical process and mechanism. Nevertheless, microbial rhodopsins exhibit remarkable diversity in both function and oligomeric states, including homodimeric channelrhodopsins^91^ and a widely distributed family of homodimeric heliorhodopsins.^92−94^ In these homodimers, the retinal chromophores are positioned similarly close to each other as in homodimeric NeoR (as shown in Figure S9), suggesting that native oligomeric structures exist that could support EET. Note that rhodopsin bistability would not even be a strict requirement for the occurrence of EET: even long-lived M-like states such as have been detected in some anion channelrhodopsins^95^ could absorb photons under intermediate to high light conditions and transfer energy. Due to the growing available genetic data the number of newly discovered microbial rhodopsins increases rapidly^5,96^ and therefore the EET process characterized in this study might prove to be of importance.

## Conclusions

Here, we demonstrated the occurrence of EET from photoexcited unprotonated RSB to protonated RSB in NeoR dimers and determined the EET rate at (200 ps)^−1^. We show that the NeoR_367_ 2A_g_^–^ excited state acts as the EET donor and NeoR_690_ acts as the EET acceptor. In NeoR_367_, light is absorbed by the 1B_u_^+^ (S3) state, which rapidly internally converts to the optically forbidden 2A_g_^–^ (S1) state in 39 fs. The theoretically predicted nπ* (S2) state^20^ does not get populated in any appreciable transient concentration during the excited-state relaxation cascade. The NeoR_367_ S1 state shows a weak stimulated emission band in the near-IR around 700 nm, which may result from mixing with an ICT state, enhancing the transition dipole moment of the S1–S0 transition and possibly facilitating the EET process. To our knowledge, this is the first time a retinal–retinal EET process has been reported in an oligomeric rhodopsin structure and might bear relevance to the function of bistable multiwavelength rhodopsin oligomers, and in particular might mediate regulatory functions at nonsaturating light intensities.

## Methods

### Protein Purification

The gene encoding NeoR from *Rhizoclosmatium globosum* (UniProtKB A0A1Y2CSJ0; residues 1–279), fused to a C-terminal StrepII tag, was cloned into the pPICZ plasmid (Thermo Fisher Scientific, Waltham, MA, USA). The protein was expressed in the methylotrophic yeast *Pichia pastoris* and purified as previously described.^7^ Briefly, cell membranes containing NeoR were solubilized in a mixture of dodecyl-maltoside (DDM; Glycon, Luckenwalde, Germany) and cholesteryl hemisuccinate (CHS; Merck, Darmstadt, Germany) at a 5:1 ratio and affinity chromatography was performed using a Strep-TactinXT 4Flow column (IBA Lifesciences GmbH, Göttingen, Germany). The eluted protein was concentrated in 50 mM Hepes pH 7.4, 100 mM NaCl and 0.02%/0.004% DDM/CHS and flash frozen in liquid nitrogen and stored at −80 °C until the spectroscopic measurements.

For cryo-electron microscopy (cryo-EM) analysis, DDM was replaced by lauryl maltose neopentyl glycol (LMNG; Anatrace Inc., Maumee, OH, USA) and proceeding the affinity chromatography the protein underwent two sequential size-exclusion chromatography (SEC) steps: first on a Superdex 200 Increase 10/300 column, followed by a Superose 6 Increase 3.2/300 column (both from Cytiva, Marlborough, MA, USA), pre-equilibrated with SEC buffer containing 50 mM Hepes pH 7.4, 100 mM NaCl and 0.02%/0.004% LMNG/CHS. Peak fractions containing NeoR were pooled and concentrated for cryo-EM analysis.

### Ultrafast TA Spectroscopy

The home-built TA setup was constructed around a 1 kHz amplified Ti/sapphire laser system (Femtopower, Spectra Physics) that served as the primary source of both the pump and the probe beam.^97^ As the actual probe beam (1/*e*^2^-diameter in focus: 50 μm) served the white-light supercontinuum generated in an argon-filled hollow-core fiber (Ultrafast Innovations). Two experiments were taken: the one presented in Figure 2 and that presented in Figure 4. For the experiment of Figure 2, the pump beam (360 nm, 20 nJ per pulse, 1/*e*^2^-diameter in focus: 85 μm) was generated in a NOPA (TOPAS, Light Conversion). In addition, the coherent/cross phase modulation artifact of the buffer was measured and subtracted from the data. The width of the IRF was 106 fs at full width, half-maximum. The timing between the pump and probe pulses was controlled by a mechanical delay line. A total of 1000 time points were recorded. The relative polarization of the pump and probe beams was set to magic angle. Both beams were chopped by optomechanical choppers allowing for shot-to-shot basis detection of the TA signal corrected for pump scattering and detector dark current. The probe transmitted through the sample was spectrally dispersed in a home-built dual-channel prism spectrometer and detected by a CCD camera (Entwicklungsbüro Stresing). The probe spectral fluctuations negatively affecting the TA signal quality were corrected using the approach described in ref (98). The sample was kept in a 0.5 mm-thick optical cell, the position of which was scanned in the transversal plane during the experiment to replenish the fresh sample. The sample was illuminated by 660 nm LED light at an intensity of 9 mW/cm^2^ to convert NeoR mostly to the NeoR_367_ state and keep it in that state. For the experiments in Figure 4, the same experimental parameters were used except that the power of the LED was manually adjusted on-the-fly to maintain the desired ratio of spectral forms present in the absorption spectrum. In addition, less time points were taken up to for speedy data acquisition. The IRF was estimated at 166 fs.

### FSRS Spectroscopy

The femtosecond-stimulated Raman spectroscopy setup is an upgraded version^6,97,99^ of the one described in our previous work.^26^ In the current design, we seeded two independent 1 kHz chirped pulse amplifiers (CPAs) with femtosecond pulses from one shared Ti: sapphire oscillator. We delayed the seed electronically and optically to trace processes beyond 6 ns. To initiate a photoreaction, we employed pulses of 240 nJ at 360 nm from the OPA driven by the Solstice amplifier focused into a 100 μm spot as the actinic pump. Actinic pulse duration was about 60 fs (full width half-maximum). Meanwhile, we focused a 1450 nm signal beam from a second OPA system on a moving CaF_2_ plate to generate a white light supercontinuum as the probe, and the probe was focused on the sample at a spot of approximately 50 μm. In the detection apparatus, the probe was split into two beams. One part was sent to a grating-based high resolution imaging spectrograph (Acton, Princeton instruments) for Raman analysis in the 750–950 nm region. The other part was directed to a prism spectrograph to obtain transient absorption spectra in the 370–1200 nm range. In both spectrographs, a 58 × 1024 pixels CCD camera (Entwicklungsbuero Stresing) was used as a linear image sensor via operation in a full vertical binning mode. Cameras were triggered from the lasers at 1 kHz and provided shot-to-shot detection. The 800 nm femtosecond pulses from the second amplifier passed through a home-built pulse shaper to create a series of frequency-locked picosecond pulses as the Raman pump, totalling 96 wavelength-shifted Raman pumps. The energy of the Raman pump was 6 μJ. We measured FSRS spectra at 34 time delays from −1.5 ps to 1.1 ns. All the experiments were taken under the magic-angle (54.7°) condition to remove the influence of orientation relaxation. To reduce the impact of photodamage in the measurement, we moved the sample in the beam at a speed of approximately 10 cm/s in a sample scanner. The sample was contained in a cuvette is 1 mm path length and kept in the NeoR_367_ state by background illumination with a red LED at a power density of 9 mW/cm^2^.

### Global and Target Analysis

The general target analysis methodology has been described in.^100^ The enormous complexity of this target analysis can only be mastered with the help of the structured problem solving environment pyGlotaran,^101^ which enables simultaneous target analysis of different groups of data (1 large TA data set and 15 different TA data sets with different background illumination, in total with 526,157 data points), linking the kinetic and the IRF properties of the data sets and thereby estimating 53 nonlinear parameters and 4319 conditionally linear parameters with the help of nonlinear least-squares. The relative precision of the estimated parameters is better than 10%. To reduce the number of free parameters the SADS of the NeoR_367_ S1 and long-lived S1 states (red and purple in Figures 4D and S5) have been assumed to be identical. In addition, in the bleach region (below 405 nm) the shapes of the S2 and hot S1 SADS (gray and blue in Figures 4D and S5) were assumed to be identical. The global analysis of the FSRS data was performed as described in.^97^

### Sample Preparation for Cryo-EM

For cryo-EM grid preparation, 3.5 μL of the purified NeoR sample was applied to glow-discharged Quantifoil R1.2/1.3 300-mesh copper grids (Quantifoil Micro Tools GmbH, Germany). Glow discharge was performed using a PELCO easiGlow device (Ted Pella, Inc.) at 15 mA for 100 s. Grids were blotted for 6 s using Whatman no. 595 filter paper (Sigma-Aldrich) in a Vitrobot Mark IV (Thermo Fisher Scientific) set to 4 °C and 100% relative humidity, with no additional blot force applied. The grids were then vitrified by rapid plunging into liquid ethane cooled by liquid nitrogen and stored in liquid nitrogen until data collection.

### Electron Microscopy Data Acquisition and Processing

Cryo-EM data were collected on a JEOL CRYO ARM 200 microscope operated at 200 kV, equipped with an in-column Omega energy filter (20 eV slit width) and a Gatan K2 Summit direct electron detector. Automated data acquisition was managed using SerialEM software at a nominal magnification of 60,000×, yielding a calibrated pixel size of 0.7981 Å/pixel. A defocus range from −0.5 to −2.0 μm was employed to optimize image contrast. Movies were recorded in counting mode as TIFF files, dose-fractionated into 42 frames over 4.2 s, with a total electron dose of approximately 45 e^–^/Å^2^ (∼1.07 e^–^/Å^2^ per frame). Real-time preprocessing, including motion correction and defocus estimation, was monitored using cryoSPARC^102^ Live (Structura Biotechnology Inc.) to ensure data quality. A total of 2391 movies were processed using RELION 5.0. Motion correction and dose-weighting were performed with MotionCor2^103^ using 5 × 5 patch alignment. Contrast transfer function (CTF) parameters were estimated with CTFFIND4.^104^

Initial particle picking was conducted on 1000 micrographs using RELION’s Blob Picker with a Laplacian-of-Gaussian filter, targeting particles between 70 and 120 Å in diameter. This yielded 645,116 particle coordinates. Extracted particles were boxed at 256 pixels and downsampled to 128 pixels to expedite processing. Two rounds of 2D classification reduced the data set to 45,861 high-quality particles.

Template-based particle picking was then performed using eight representative 2D class averages, resulting in 1,135,263 particles. After multiple rounds of 2D classification to eliminate false positives and poorly aligned particles, 511,623 particles were selected for 3D reconstruction. An ab initio 3D model was generated using a mask diameter of 160 Å in three classes to capture initial structural features. The best-resolved class was further refined through 3D classification in RELION,^105^ employing Blush Regularization, which reduced the data set to 230,814 particles.

Final 3D refinement was conducted under C1 symmetry, yielding a cryo-EM map at a global resolution of 4.80 Å, determined by the gold-standard Fourier shell correlation (FSC) at the 0.143 threshold. Postprocessing steps included map sharpening and local resolution estimation. The map’s interpretability was further enhanced using DeepEMhancer,^106^ facilitating downstream model building. The relative orientation of two NeoR monomers obtained from alphafold2,^28^ with retinal position taken from structural superposition with the crystal structure of Rhodopsin-Phosphodiesterase^29^ (RhoPDE, PDB: 7CJ3) was fitted into the map in ChimeraX 1.8,^107^ (UCSF Resource for Biocomputing). Structures were visualized in Pymol (The PyMOL Molecular Graphics System, Version 3.0 Schrödinger, LLC.).

## Supporting Information Available

Supplemental figures for raw TA data and global analysis result and residual, raw FSRS kinetics and global fit result, cryo-EM single particle analysis, analysis of in situ absorption spectra, TA traces under various illumination conditions and their target analysis, target analysis kinetic schemes, population profile, SADS, raw data, fit result and residual from target analysis of TA data, retinal–retinal arrangements in various homodimeric microbial rhodopsins, structure-bases FRET calculation on the NeoR homodimer (PDF)
