# Supplementary material for: Retinal to Retinal Energy Transfer in a Bistable Microbial Rhodopsin Dimer
Source: J Am Chem Soc. 2025 Apr 17;147(17):14468–80. doi: 10.1021/jacs.5c01276 (PMC12046560; doi:10.1021/jacs.5c01276)
Supplement: Supplementary file 1 — ja5c01276_si_001.pdf [file ja5c01276_si_001.pdf]

Supporting information to

Retinal to Retinal Energy Transfer in a Bistable Microbial Rhodopsin Dimer

Ivo H.M. van Stokkum[1], Jakub Dostal[2], Thanh Nhut Do[1], Lifei Fu[3], Gregor Madej[3], Christine Ziegler[3], Peter Hegemann[4], Miroslav Kloz[2], Matthias Broser[4]*, John T.M. Kennis[1]*

[1] Department of Physics and Astronomy, Faculty of Science, Vrije Universiteit Amsterdam, De Boelelaan 1081, 1081 HV, Amsterdam, The Netherlands.

[2] ELI Beamlines Facility, The Extreme Light Infrastructure ERIC, Za Radnicí 835, 25241 Dolní Břežany, Czech Republic.

[3] Department of Structural Biology/Biophysics II, University of Regensburg, Regensburg, Germany

[4] Institut für Biologie, Experimentelle Biophysik, Humboldt-Universität zu Berlin, Invalidenstr. 42, D-10115 Berlin, Germany.

Corresponding authors, matthias.broser@hu-berlin.de, j.t.m.kennis@vu.nl

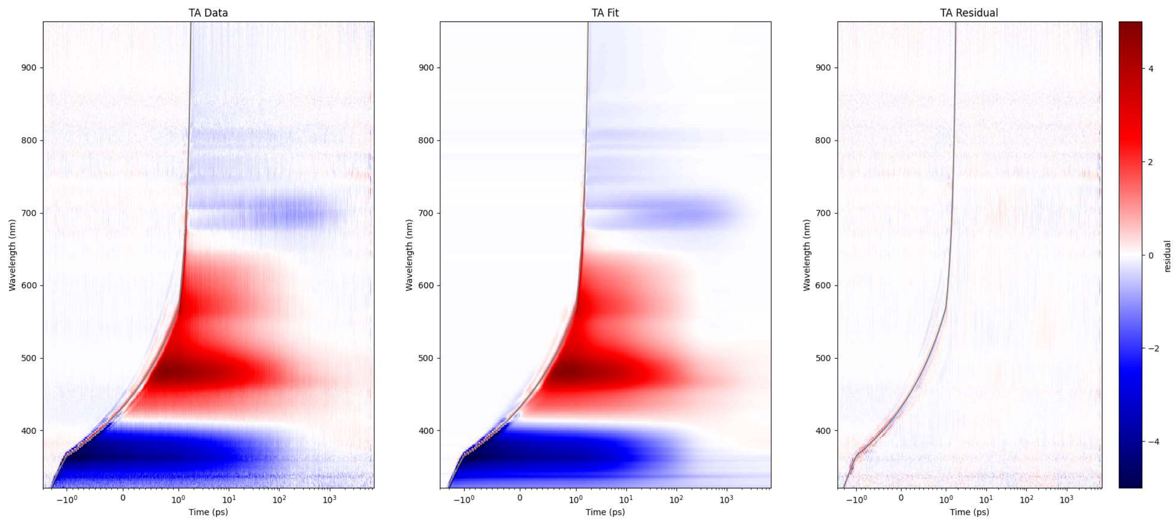

Figure S 1. Global analysis of the 6 ns TA experiment used for Figure 2 (in mOD), note the qualitative and quantitative agreement. From left to right: data, fit and residual. In grey the estimated dispersion curves (the location of the maximum of the IRF). Note that the time axis is linear from -1 to 1 ps and logarithmic elsewhere.

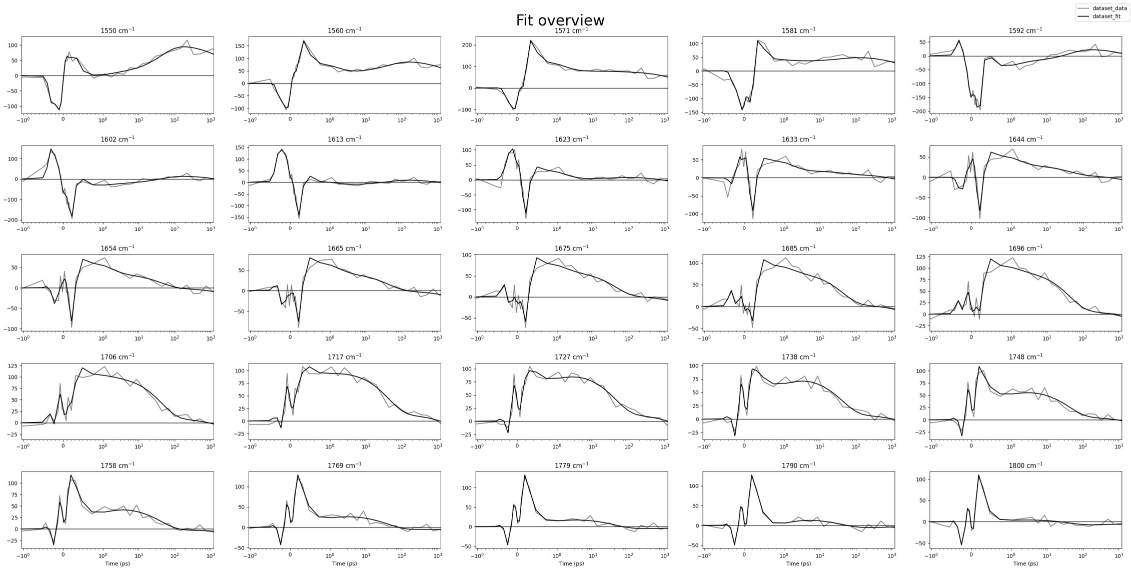

Figure S2. Fit quality at 25 selected wavenumbers between 1550 and 1800 cm^{-1} and till 1 ns of FSRS of NeoR$_{367}$ (grey), fit lines in black. Note that the time axis is linear from -1 to 1 ps and logarithmic thereafter.

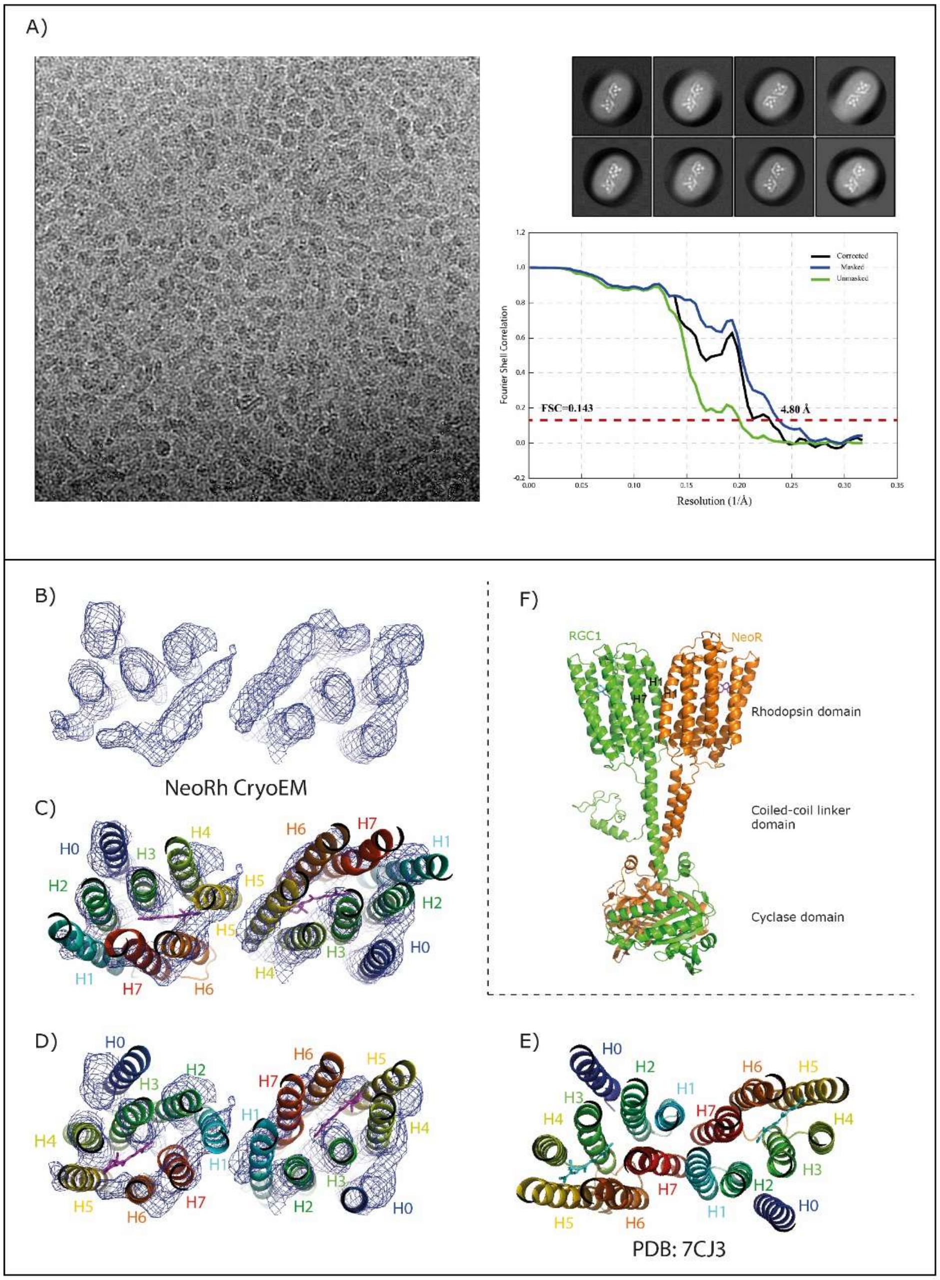

Figure S3. Cryo-EM single-particle analysis of the NeoR dimer.

- *A) Representative cryo-EM micrograph, reference-free 2D class averages and final 3D reconstruction map FSC curve*
- *B) CryoEM map drawn as mesh at a contour level of 5*
- *C) Fitted NeoR structure based on an Alphafold2 model*
- *D) NeoR model as in B), but fitted with helix H1 at the C2-axis*
- *E) Crystal structure of homodimeric RhoPDE[1]*
- *F) Overall arrangement of a full-length heterodimeric RGC1/NeoR complex as derived from Alphafold2*

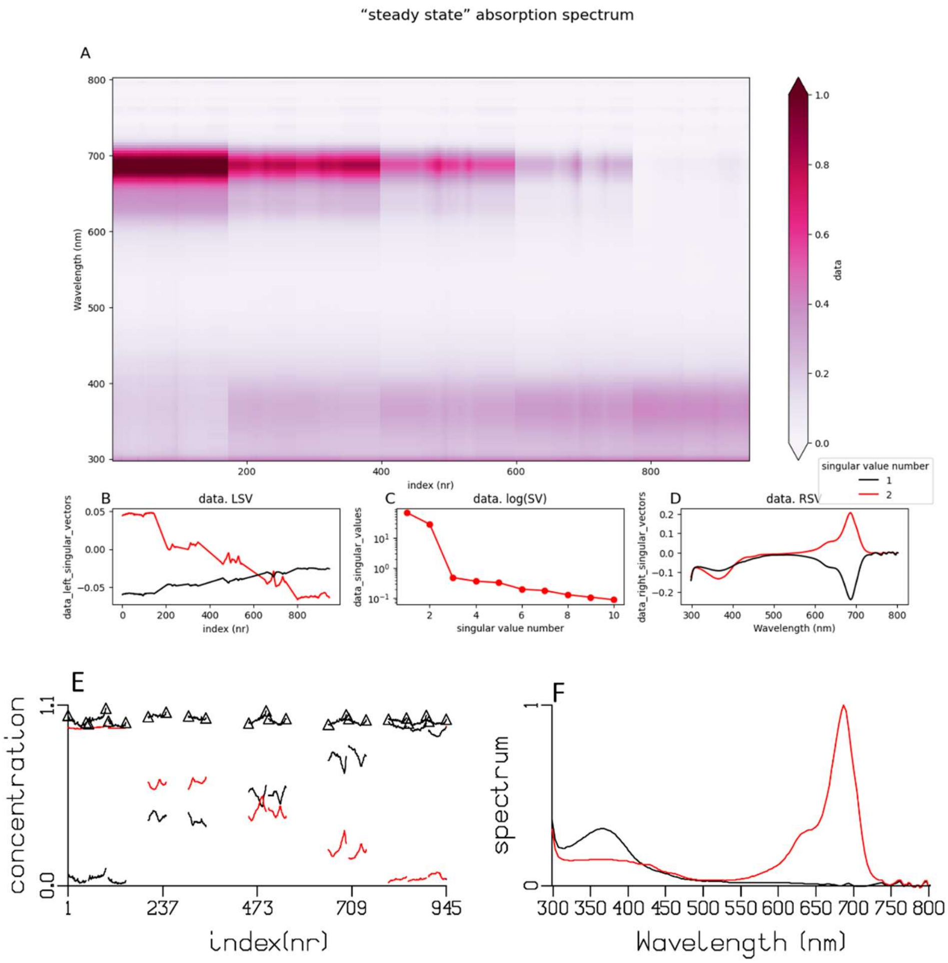

Figure S4. Decomposition of the "steady state" absorption spectrum measured in situ during the experiments (A, in OD). (B-D) The singular value decomposition of the matrix of the data: (B) first two left singular vectors, (D) first two right singular vectors, (C) screeplot of the singular values, indicating the presence of two components. The abscissa in A,C,E indicates the index (nr) of the time gated absorption spectra, which are a linear combination of the two component spectra. These component spectra are depicted in (F). Key: black, NeoR$_{367}$ state and red, NeoR$_{690}$ state. Concentrations (E): in the first three scans no led was used, thus almost pure NeoR$_{690}$ state with concentration almost 1 (experiment 1). In the next two scans (experiment 2) ≈25% of NeoR$_{690}$ was converted to NeoR$_{367}$. Next, ≈50% (experiment 3), ≈75% (experiment 4) and almost all of NeoR$_{690}$ (experiment 5) was converted to NeoR$_{367}$. Note that the NeoR$_{690}$ concentration (red) decreases in steps, whereas the NeoR$_{367}$ concentration (black) increases in steps. The sum, indicated by the triangles, is almost constant. Note that the UV absorption of the fitted NeoR$_{690}$ spectrum appears overestimated, which may result from light scattering at short wavelengths. Therefore, we consider the NeoR$_{690}$ concentration of experiment 1 (almost pure NeoR$_{690}$) uncertain and the value that results from the analysis of Fig. S5 (83% concentration, see the Table in Fig. 4) more reliable.

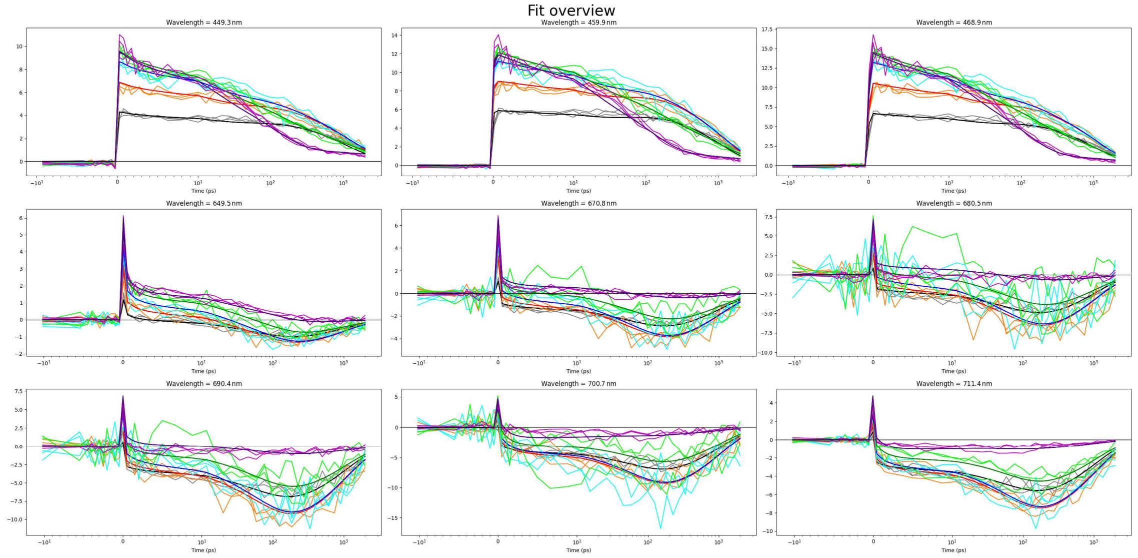

Figure S5. Transient absorption (in units of mOD) of NeoR in five conditions (3 scans each) at nine selected wavelengths (indicated in the ordinate label). See Figure 4 for color code and fractions UV-UV, Red-Red and UV-Red. Scaling numbers in the analysis are experiment 1, magenta, 1.21; experiment 2, green, 1.23; experiment 3, cyan, 1.28; experiment 4, orange, 1.12, experiment 5, grey, unscaled. The overall rms error of the fit was 0.22 mOD. The scaling numbers constitute a correction on the estimated fractions that follow from the analysis of Fig. S4. The fact that they are close to 1 gives confidence that the applied kinetic scheme is valid. Black, red, blue, dark green and purple lines indicate the target analysis fit. Note that the time axis is linear until 10 ps and logarithmic thereafter.

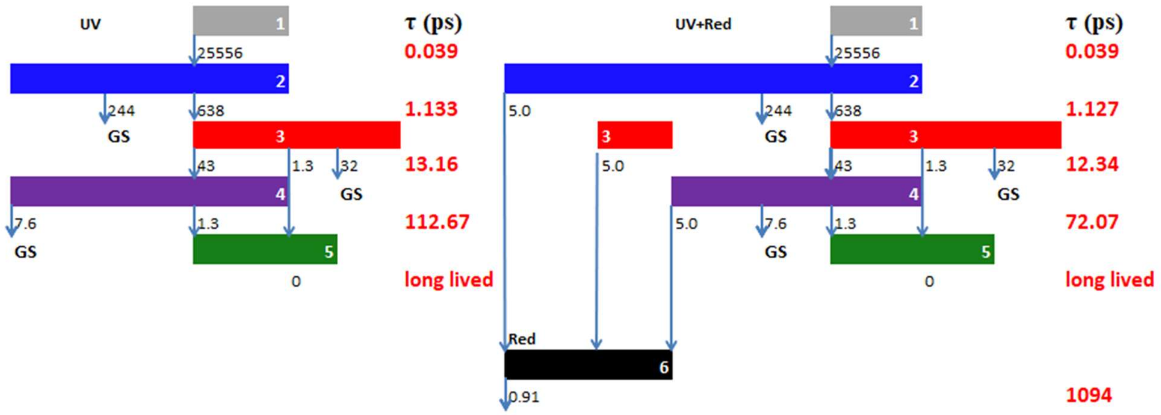

Figure S6. Kinetic schemes for the dimer state (UV-UV) (left) and for the Dimer state UV-Red (right). The Red-Red dimer state is taken into account as a directly excited fraction of NeoR$_{690}$. The color code is identical to that of Fig. 4.*

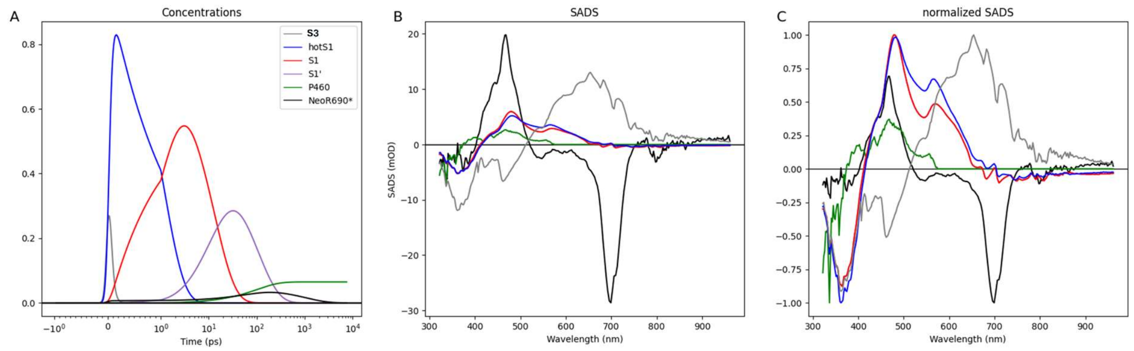

Figure S7. Populations (A), SADS (B, in mOD) and normalized SADS (C) estimated from the TA data of Figure 2 (fit shown in Figure S 8).

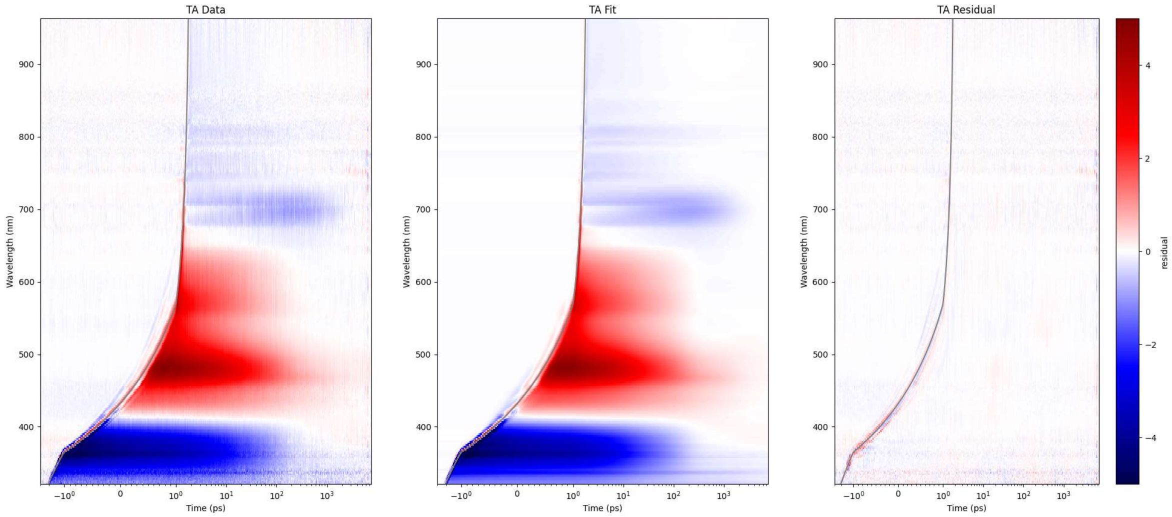

Figure S8. Target analysis of the 6 ns TA experiment used for Figure 2 according to the kinetic model of Figure S6 (in mOD), note the qualitative and quantitative agreement. From left to right: data, fit and residual. In grey the estimated dispersion curves (the location of the maximum of the IRF). Note that the time axis is linear from -1 to 1 ps and logarithmic elsewhere.

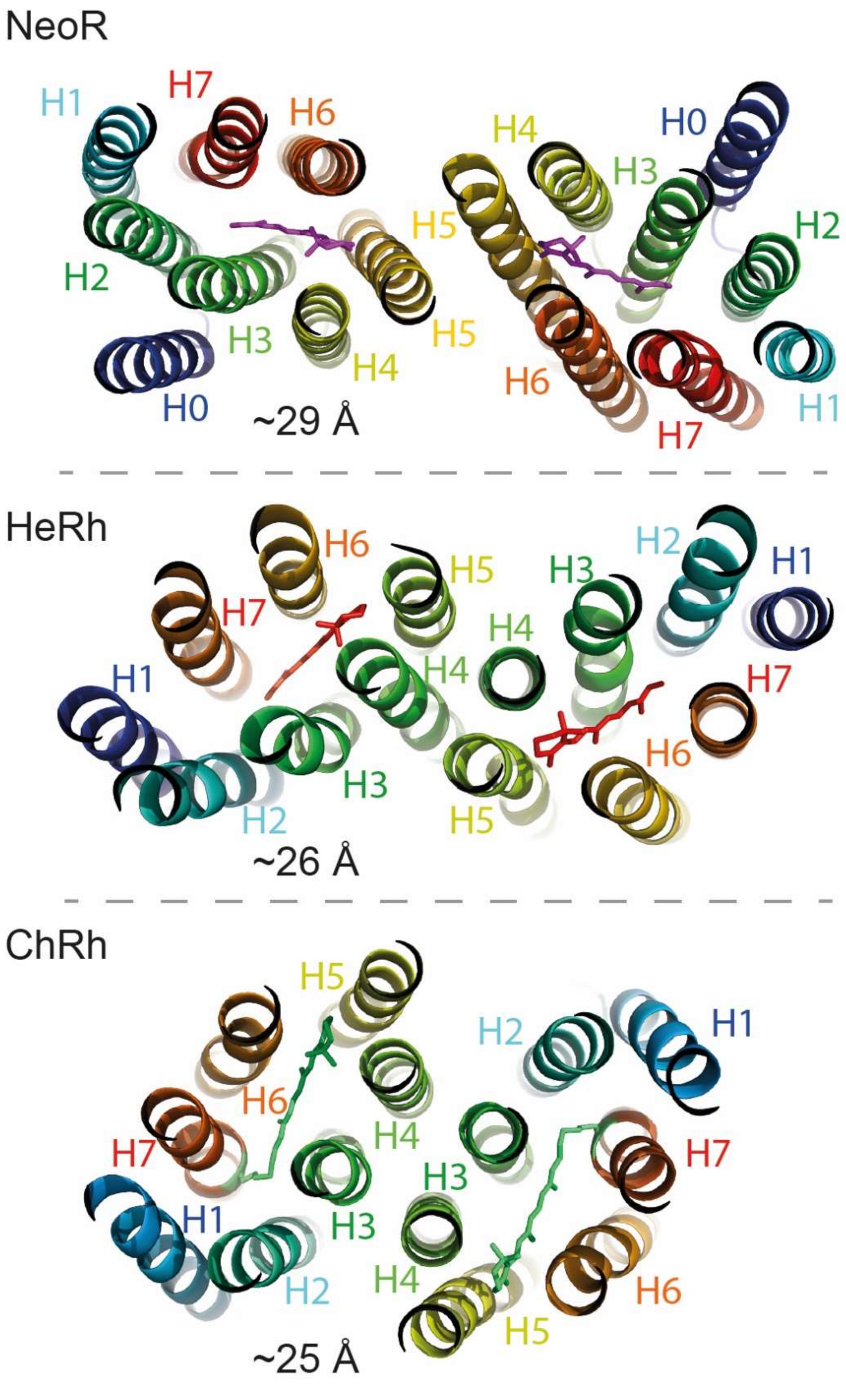

Figure S9: retinal-retinal arrangements in microbial rhodopsin homodimers: NeoR, Heliorhodopsin (HeRh; PDB: 6SU3) [2] and Channelrhodopsin Chrimson (ChRh; PDB: 5ZIH[3] with center-to-center distances indicated.

Structure-based Forster calculation on the NeoR homodimer

We performed a structure-based Förster calculation to estimate the excitation energy transfer (EET) rate between the UV absorbing ($NeoR_{367}$) and red absorbing ($NeoR_{690}$) RSB in the NeoR dimer. The calculation routine is similar as the one used by Kleima et al.[4] The coordinates of all atoms of the RSBs are extracted from the Cryo-EM structure resolved at 4.8-Å resolution. We did not take into account the 7-cis conformation in the $NeoR_{367}$ state.

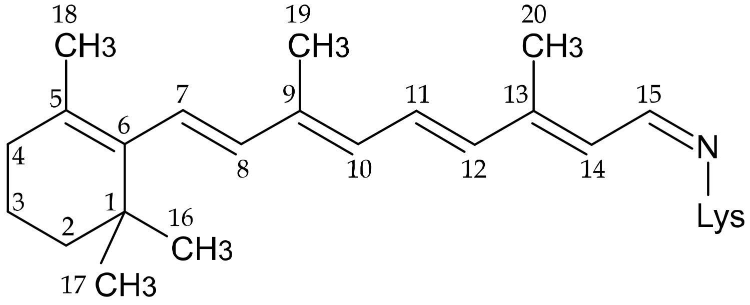

Figure S10. Chemical structure of the retinal Schiff base (RSB) with all C atoms are numbered according to the conventional nomenclature.

The chemical structure of the RSB is shown in Figure S10. The transition dipole moment of each RSB is defined as the vector connecting the N atom to the C atom numbered 5. The center of RSB chromophore is determined as the center of mass of all heavy atoms (C and N atoms) shown in Figure S10. Refractive index of the protein medium is set as n = 1.6.[4]

With the above structural definitions, the Förster EET rate is calculated as

$$k_{EET} = 8.8 \times 10^{17} \times \frac{\kappa^2}{R^6} \times \frac{k_r^D}{n^4} \int \frac{\epsilon_A(\nu) F_D(\nu)}{\nu^4} \mathrm{d}\nu$$

where, k_{EET} is the Förster EET rate in ps^{-1}, and R is the inter-chromophoric distance in nm defined as the distance between two centers-of-mass of two RSB. k_r^D is the radiative decay rate of the donor, in this case the deprotonated RSB. The orientation factor κ is calculated as

$$\kappa = \overrightarrow{\mu_A} \cdot \overrightarrow{\mu_D} - 3(\overrightarrow{\mu_A} \cdot \vec{R})(\overrightarrow{\mu_D} \cdot \vec{R})$$

$\overrightarrow{\mu_A}$, $\overrightarrow{\mu_D}$, and $\vec{R}$ are the unit vectors of transition dipole moments of the acceptor, the donor, and the interchromophoric distance, respectively.

The integral represents spectral overlap on the frequency scale ν of cm^{-1} between the absorption cross-section $\epsilon_A(\nu)$ normalized with the peak of extinction coefficient at 690 nm of 129,000 $M^{-1}cm^{-1}$ [5] and the fluorescence spectrum $F_D(\nu)$ extracted from Bachilo et al.[6], fitted with two Gaussian functions, and area normalized on the frequency scale of cm^{-1}.

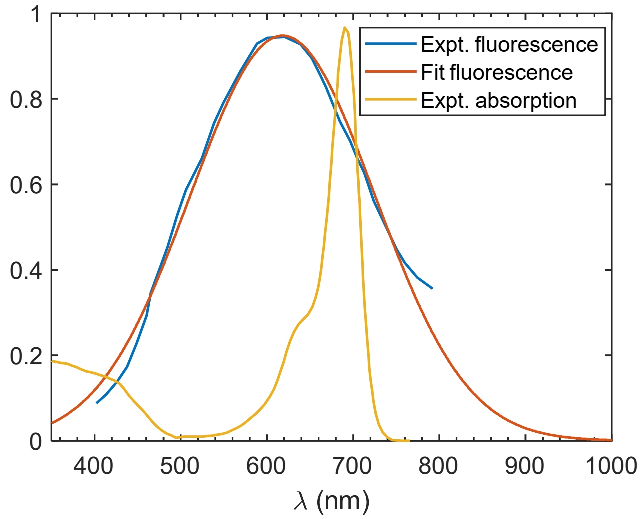

Figure S11. Fluorescence spectrum of unprotonated RSB measured in ethanol (blue line) extracted from Bachilo et al. overlaid with the Gaussian fit (orange line). The absorption spectrum of NeoR is reported by Broser et al. (yellow line).

The absorption spectrum is reported in NeoR rhodopsin by Broser et al.[5] (yellow line), the fluorescence spectrum of deprotonated RSB in ethanol is measured by Bachilo et al.[6] (blue line) are shown together with the Gaussian fit of the fluorescence spectrum (orange line) in Figure S2. These spectra show a significant spectral overlap between the emission spectrum of unprotonated RSB and absorption spectrum of NeoR, indicating that the EET may occur (if other Förster EET conditions are favored).

With the parameters described above and an assumed radiative rate of (250 ns)$^{-1}$ for unprotonated RSB in ethanol,[6] the Förster EET rate is calculated as k_{EET} = 0.001 ps^{-1} or the corresponding time-constant is 1.0 ns.

Based on the experimental observations, the calculated results of k_{EET} is rather slow, 1 ns vs. the 200 ps experimental result. The deviation may be due to the fact that our calculation is rather crude with various approximations (slow radiative decay time of 250 ns for unprotonated RSB in ethanol, the fluorescence spectrum of unprotonated RSB in solution, the 1.6 value for the refractive index). To estimate the effects of radiative decay time-constant, we repeated the calculation with the value of $1/k_r^D$ varied from 10 ns to 100 ns and the results are shown in Figure S12. The results show that to yield the EET time-constant of 200 ps, the radiative decay time should be around 50 ns, a factor of 5 faster than the value of free RSB in ethanol.

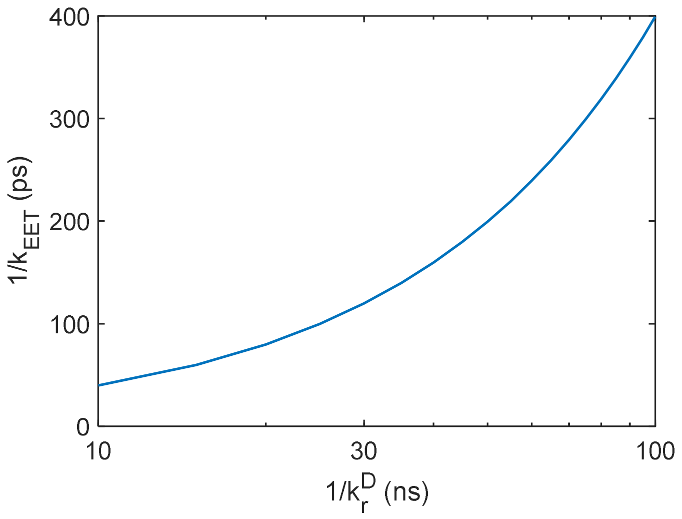

Figure S12. The variation of k_{EET} obtained by varying the value of radiative decay time constant from 10 ns to 100 ns.

References

(1) Ikuta, T.; Shihoya, W.; Sugiura, M.; Yoshida, K.; Watari, M.; Tokano, T.; Yamashita, K.; Katayama, K.; Tsunoda, S. P.; Uchihashi, T.; et al. Structural insights into the mechanism of rhodopsin phosphodiesterase. *Nature Communications* **2020**, *11* (1). DOI: 10.1038/s41467-020-19376-7.
(2) Kovalev, K.; Volkov, D.; Astashkin, R.; Alekseev, A.; Gushchin, I.; Haro-Moreno, J. M.; Chizhov, I.; Siletsky, S.; Mamedov, M.; Rogachev, A.; et al. High-resolution structural insights into the heliorhodopsin family. *Proceedings of the National Academy of Sciences of the United States of America* **2020**, *117* (8), 4131-4141. DOI: 10.1073/pnas.1915888117.
(3) Oda, K.; Vierock, J.; Oishi, S.; Rodriguez-Rozada, S.; Taniguchi, R.; Yamashita, K.; Wiegert, J. S.; Nishizawa, T.; Hegemann, P.; Nureki, O. Crystal structure of the red light-activated channelrhodopsin Chrimson. *Nature Communications* **2018**, *9*. DOI: 10.1038/s41467-018-06421-9.
(4) Kleima, F. J.; Hofmann, E.; Gobets, B.; van Stokkum, I. H. M.; van Grondelle, R.; Diederichs, K.; van Amerongen, H. Forster excitation energy transfer in peridinin-chlorophyll-<i>a</i>-protein. *Biophysical Journal* **2000**, *78* (1), 344-353. DOI: 10.1016/s0006-3495(00)76597-0.
(5) Broser, M.; Spreen, A.; Konold, P. E.; Peter, E.; Adam, S.; Borin, V.; Schapiro, I.; Seifert, R.; Kennis, J. T. M.; Sierra, Y. A. B.; et al. NeoR, a near-infrared absorbing rhodopsin. *Nature Communications* **2020**, *11* (1). DOI: 10.1038/s41467-020-19375-8.
(6) Bachilo, S. M.; Bondarev, S. L.; Gillbro, T. Fluorescence properties of protonated and unprotonated Schiff bases of retinal at room temperature. *Journal of Photochemistry and Photobiology B-Biology* **1996**, *34* (1), 39-46. DOI: 10.1016/1011-1344(95)07271-3.
